# Ginsenoside Rg_5_ Activates the LKB1/AMPK/mTOR Signaling Pathway and Modifies the Gut Microbiota to Alleviate Nonalcoholic Fatty Liver Disease Induced by a High-Fat Diet

**DOI:** 10.3390/nu16060842

**Published:** 2024-03-15

**Authors:** Yingying Shi, Jianbo Chen, Di Qu, Qiang Sun, Yang Yu, Hao Zhang, Zhengbo Liu, Jiyue Sha, Yinshi Sun

**Affiliations:** 1 College of Chinese Medicinal Materials, Jilin Agricultural University, Changchun 130118, China; syingying0522@163.com (Y.S.); sunqiang133543@163.com (Q.S.); 13103881091@163.com (H.Z.); 2 Institute of Special Wild Economic Animals and Plants, Chinese Academy of Agricultural Sciences, Changchun 130112, China; chenjianbo00882@126.com (J.C.); qudi@caas.cn (D.Q.); m15941105393@163.com (Y.Y.); 18043213739@163.com (Z.L.)

**Keywords:** ginsenoside Rg_5_, nonalcoholic fatty liver disease, fecal microbiota transplantation, LKB1/AMPK/mTOR signaling pathway

## Abstract

The primary objective of this investigation was to elucidate the manner in which ginsenoside Rg_5_ (Rg_5_) ameliorates nonalcoholic fatty liver disease (NAFLD) via the modulation of the gut microbiota milieu. We administered either a standard diet (ND) or a high-fat diet (HFD), coupled with 12-week treatment employing two distinct doses of Rg_5_ (50 and 100 mg/kg/d), to male C57BL/6J mice. In comparison to the HFD cohort, the Rg_5_-treated group demonstrated significant enhancements in biochemical parameters, exemplified by a substantial decrease in lipid concentrations, as well as the reduced expression of markers indicative of oxidative stress and liver injury. This signifies a mitigation of hepatic dysfunction induced by an HFD. Simultaneously, Rg_5_ demonstrates the capacity to activate the LKB1/AMPK/mTOR signaling pathway, instigating energy metabolism and consequently hindering the progression of NAFLD. Furthermore, we underscored the role of Rg_5_ in the treatment of NAFLD within the gut-microbiota-liver axis. Analysis via 16S rRNA sequencing unveiled that Rg_5_ intervention induced alterations in gut microbiota composition, fostering an increase in beneficial bacteria, such as *Bacteroides* and *Akkermansia*, while concurrently reducing the relative abundance of detrimental bacteria, exemplified by *Olsenella*. Furthermore, employing fecal microbiota transplantation (FMT) experiments, we observed analogous outcomes in mice subjected to fecal bacterial transplants, providing additional verification of the capacity of Rg_5_ to mitigate NAFLD in mice by actively participating in the restoration of gut microbiota via FMT. Drawing from these data, the regulation of the gut microbiota is recognized as an innovative strategy for treating or preventing NAFLD and metabolic syndrome. Consequently, these research findings suggest that Rg_5_ holds promise as a potential therapeutic agent for NAFLD management.

## 1. Introduction

Nonalcoholic fatty liver disease (NAFLD) is a multi-systemic metabolic disorder [1]. As per reports, NAFLD distinguishes itself as the most prevalent hepatic disorder worldwide, impacting more than a third of the global population and representing a substantial menace to human health [2]. The pathophysiology of NAFLD is highly complex and includes a range of factors such as dyslipidemia, oxidative stress, inflammatory response, metabolic dysregulation, altered gut microbiota, and genetics [3,4,5]. It is widely recognized that gut microbes exert a substantial influence on human health, participating in numerous physiological processes either directly or indirectly [6,7,8]. More specifically, this encompasses immune regulation, along with the modulation of hormones and metabolites. Disruptions in the intestinal microbial ecosystem possess anti-obesity characteristics and may contribute to the development of various endocrine system disorders, including diabetes and thyroid-related issues [9,10]. The production of short-chain fatty acids (SCFAs) is primarily attributed to fermentation by the gut microbiota in the large intestine. The most prevalent SCFAs in the gastrointestinal tract include acetate, propionate, and butyrate, collectively constituting a minimum of 95% of the total. They actively contribute to maintaining the integrity of the intestinal barrier, controlling appetite, and modulating immune functions [11]. The gut microbiota can produce essential amino acids, vitamins, and other metabolites that assist in the prevention of pathogen colonization, along with SCFAs.

Ginsenoside Rg_5_ (Rg_5_) is a rare ginsenoside with high bioavailability, showcasing various advantageous biological properties [12]. Nevertheless, the impact of Rg_5_ on NAFLD and its underlying mechanisms remains unclear. Furthermore, fecal microbiota transplantation (FMT) has garnered attention as a focus of recent research, displaying promising outcomes in the treatment of various conditions, including obesity [13]. Therefore, the aim of this study was to explore the effect of Rg_5_ on NAFLD, with special attention paid to elucidating the role of gut microbiota and providing new insights into the potential target and mechanism of Rg_5_ as an NAFLD therapeutic agent.

## 2. Materials and Methods

### 2.1. Materials

We purchased Rg_5_ with a purity > 98% (item number 186763-78-0), made in China, from Changchun Yuanda Biotechnology Co. Ltd., Changchun, China. We obtained 10% and 60% kcal fat calorie high-fat mouse foods (U.S. item number 112252) from Dyets Biotechnology (Wuxi, China) Co., Ltd.; glucose (item number DE0149) was purchased from Beijing BioDee Biotechnology Co., Ltd., Beijing, China; insulin (item number Y0001717) was acquired from Sigma Aldrich (Shanghai, China) Trading Co., Ltd. Assay kits for alanine aminotransferase (GPT/ALT) (item number C009-2-1), aspartate aminotransferase (GOT/AST) (item number C010-2-1), catalase from micrococcus lysodeiktic (CAT) (item number A007-1-1), superoxide dismutase (SOD) (item number A001-1-2), malondialdehyde (MDA) (item number A003-1-2), and glutathione peroxidase (GSH-Px) (item number A005-1-2) were purchased from Nanjing Jiancheng Haihao Biotechnology Co., Ltd., Nanjing, China. We acquired vancomycin (V871983-1 g); neomycin sulfate (N814740-25 g); metronidazole (M813526-25 g); and ampicillin (A830931-5 g), Macklin brand, from Beijing Essun Huitong Technology Co., Ltd., Beijing, China. We obtained metaphosphoric acid of analytical purity from Sinopharm Chemical Reagent Co., Ltd., Shanghai, China. We procured ethyl butyric acid of analytical purity from Sinopharm Chemical Reagent Co., Ltd., (Shanghai, China). Furthermore, we obtained acetic acid (purity of 99.9%, CAS NO. 64-19-7), propionic acid (purity of 99.9%, CAS NO. 79-09-4), butyric acid (purity of 99.9%, CAS NO. 107-92-6), isobutyric acid (purity of 99.8%, CAS NO. 79-31-2), valeric acid (purity of 99.1%, CAS NO. 109-52-4), isovaleric acid (purity of 99.8%, CAS NO. 503-74-2), and hexanoic acid (purity of 99.9%, CAS NO. 142-62-1) from Shanghai Acmec Biochemical Technology Co., Ltd., Shanghai, China, using the BCA protein assay kit (P0009, Beyotime, Shanghai, China).

### 2.2. Animals

A total of 70 eight-week-old male C57BL/6J mice (18–22 g) were purchased from Liaoning Changsheng Biotechnology Co., Ltd., Changchun, China, with certificate number SCXK (Liao) 2020-0001. They were kept in an animal care facility maintained at a constant temperature of 40 °C, with a relative humidity of 50–60% and a 12 h light/12 h dark cycle. Animals were provided with free access to food and water. The animal program was approved by the Institutional Animal Care Committee of the Chinese Academy of Agricultural Sciences and complied with the Animal Management Rules of the Ministry of Health of the People’s Republic of China.

Donor mice: NAFLD mice model of 40 male C57BL/6J mice. All mice were acclimatized for 1 week and then randomly divided into four groups, namely the normal diet group (ND, *n* = 10), the high-fat diet group (HFD, *n* = 10), the low-dose group of HFD with Rg_5_ (50 mg/kg/d, *n* = 10), and the high-dose group of HFD with Rg_5_ (100 mg/kg/d, *n* = 10).

Recipient mice: FMT mice model of 30 male C57BL/6J mice. All mice were acclimatized for one week and then randomly divided into three groups: the model group (HFD →Model) (*n* = 10), the NR group (natural recovery) (*n* = 10), and the Rg_5_ group (HFD + Rg_5_→Rg_5_, *n* = 10). All mice were fed with 60% kcal chow and given access to autoclaved distilled water containing 4 antibiotics for the first month and normal autoclaved distilled water for the second month.

The ND group was fed with a normal diet, while the HFD group, HFD + Rg_5_ (50 mg/kg) group, and HFD + Rg_5_ (100 mg/kg) group were fed an HFD for three months; the components of the normal diet and the HFD are listed in the Supplementary File (Tables S1 and S2).

### 2.3. Fecal Microbiota Transplantation

Mice from the HFD and HFD + Rg_5_ (50 mg/kg/d) groups were used as donor mice and kept in an optimal environment. Fresh fecal samples were collected daily to perform FMT experiments. Fecal samples from each group of donor mice were collected and diluted 1:5 with sterile PBS. The samples were then ground up and filtered through a 70 mm filter. The resulting supernatant was collected for further use [14]. C57BL/6J male recipient mice were fed with HFD in month 1, and a fresh mixture of antibiotics (ampicillin 1 g/L, vancomycin 0.5 g/L, neomycin 1 g/L, and metronidazole 1 g/L) was added to sterile water for the recipient mice [15]. After completing the antibiotic treatment, normal sterile water was used instead of the previously mixed antibiotic water, starting from the second month. Recipient mice were then tube-fed via the daily administration of microbiota from donor mice. After two months of treatment, the animals were euthanized, and serum and tissues were collected.

### 2.4. Oral Glucose Tolerance Test (OGTT) and Insulin Tolerance Test (ITT) Iochemical Parameter Analysis

Mice were subjected to OGTT and ITT after 12 weeks of treatment. Mice fasted for 6 h before the experiment and were given 50% (*w*/*v*) D-glucose (2 g/kg/BW) solution via gavage, and tail vein blood was collected at different time points after fasting. Blood glucose levels were measured using a sinoheart blood glucose meter (China Sinocare Biosensor Co., Ltd., Changsha, China), and then the area under the curve (AUC) was calculated to quantify the cumulative change in glycemic response. In the insulin tolerance test, mice were fasted for 6 h before commencing the experiment. Then mice were intraperitoneally injected with 0.75 U/kg of insulin, blood samples were collected from the tail vein at different time points after insulin injection, blood glucose was measured, and finally the area under the curve (AUC) was calculated [16].

### 2.5. Biochemical Parameter Analysis

Total cholesterol (TC), triglyceride (TG), low-density lipid cholesterol (LDL-C), aspartate aminotransferase (AST), and almandine aminotransferase (ALT), catalase (CAT), glutathione peroxidase (GSH-Px), malondialdehyde (MDA), and superoxide dismutase (SOD), all indices of serum and liver tissues were assayed using the corresponding commercial kits (manufactured by Jiancheng, Nanjing, China).

### 2.6. Histological Analysis

Following the standard protocol, the liver and colon tissues of mice were fixed with 4% paraformaldehyde, dehydrated, and embedded in paraffin wax. Then, 4 μm-thick tissue sections were prepared and mounted onto glass slides. The liver and colon tissues were stained with hematoxylin and eosin (H&E) and Oil Red O. Finally, the images were captured using a light microscope and then subjected to analysis. Images were acquired at 200× and 400× magnification under a microscope. All data were quantified using ImageJ software version 1.54b (National Institutes of Health, Bethesda, MD, USA).

### 2.7. Western Blotting

Total protein was extracted from liver tissue and colon tissue, and BCA protein content was determined. Protein was denatured at 95 °C for 5 min, separated via polyacrylamide gel electrophoresis, and then transferred to a polyvinylidene fluoride membrane (PVDF). The membrane was blocked with 5% skim milk for 1 h at room temperature and then mixed with ZO-1 (1:500; ab96587; abcam, Cambridge, UK), Occludin (1:1000; ab216327; abcam, Cambridge, UK), Claudin-1 (1:1000; ab307692; abcam, Cambridge, UK) and GAPDH (1:10,000; ab8245; abcam, Cambridge, UK) overnight at 4 °C, as well as with LKB1 (1:1000; ab15095; abcam Cambridge, UK), AMPK (1:1000; ab32047; abcam, Cambridge, UK), p-AMPK (1:1000; Ab133448; abcam Cambridge, UK), mTOR (1:10,000; ab32047; abcam Cambridge, UK), p-mTOR (1:10,000; ab109268; abcam Cambridge, UK), and β-actin (1:1000; 4970S; CST, Danvers, ME, USA). After three washes with PBST, the membrane was then incubated with horseradish peroxidase-conjugated anti-rabbit and anti-mouse secondary antibodies (1:10,000 dilution) for 1 h at room temperature, respectively. Protein bands were imaged using ECL kits (Merck Millipore, Darmstadt, Germany), and we finally quantified band intensity using Image J software version 1.54b (National Institutes of Health, Bethesda, MD, USA).

### 2.8. 16S rRNA Sequencing

The feces of each group of mice were collected under aseptic conditions, and 0.2 g of fecal samples was sent to Shanghai Personal Biotechnology Co., Ltd. (Shanghai, China), for the detection of intestinal microorganisms using 16S rRNA analysis. For pre-treated samples, the Mag-Bind Soil DNA Kit (Catalog M5635-02) from OMEGA was used for extraction. For the extracted DNA, 0.8% agarose gel electrophoresis was performed to determine its molecular size, and the DNA was quantified using Nanodrop. We selected the 16S rRNA V3-V4 region for designing polymerase chain reaction (PCR) amplification primers. The forward primer was ACTCC-TACGGGAGGCAGCA and the reverse primer was GGACTACHVGGGTWTCTAAT. Illumina NovaSeq platform for paired-end sequencing. PCR products were quantified using the Quant-iT PicoGreen dsDNA Assay Kit on a Microplate reader (BioTek, FLx800), Invitrogen, Carlsbad, CA, USA. The products were then mixed based on the desired amount of data for each sample, and library construction was performed. Microbiome biology information was analyzed using QIIME version 2019. 4 [17]. The Greengenes database was utilized to acquire the classification data associated with every ASV [18]. We utilized R packages (version 3.6.1) and QIIME2 software (version 2022.11) for data processing tasks, such as taxonomic composition analysis. Biomarkers of differential abundance between groups were further investigated using linear discriminant analysis effect size (LEfSe) analysis and random forest analysis. Histograms were used to display the distribution of LDA values, highlighting significantly enriched species and their significance. The metabolic functions of microflora were predicted using PICRUSt2 (Phylogenetic Investigation of Communities by Reconstruction of Unobserved States) on MetaCyc and KEGG databases.

### 2.9. Detection of SCFAs Content in Mouse Feces

Gas chromatography/mass spectrometry (GC-MS) was used to determine SCFAs in mouse fecal content, including acetic acid, propionic acid, butyric acid, valeric acid, isobutyric acid, and isovaleric acid. SCFAs concentrations in mouse feces are reported in mmol/L. Sample pretreatment and the preparation of reagents were conducted, taking into account similar methods described in the literature [19,20].

### 2.10. Statistical Analysis

All data are shown as mean ± SD. To compare the two groups, a two-tailed Student’s *t*-test was used. Multiple comparisons were statistically assessed using one-way analysis of variance (ANOVA) and post hoc Tukey tests. In all cases, *p* < 0.001, *p* < 0.01, or *p* < 0.05 were considered to be statistically significant. Statistical graphs were created using the GraphPad Prism software package, version 8 (GraphPad, CA, San Diego, CA, USA).

## 3. Results

### 3.1. Effects of Rg_5_ Intervention on Body Weight, Food Intake, and Organ Indices in NAFLD Mice

**Figure 1:**
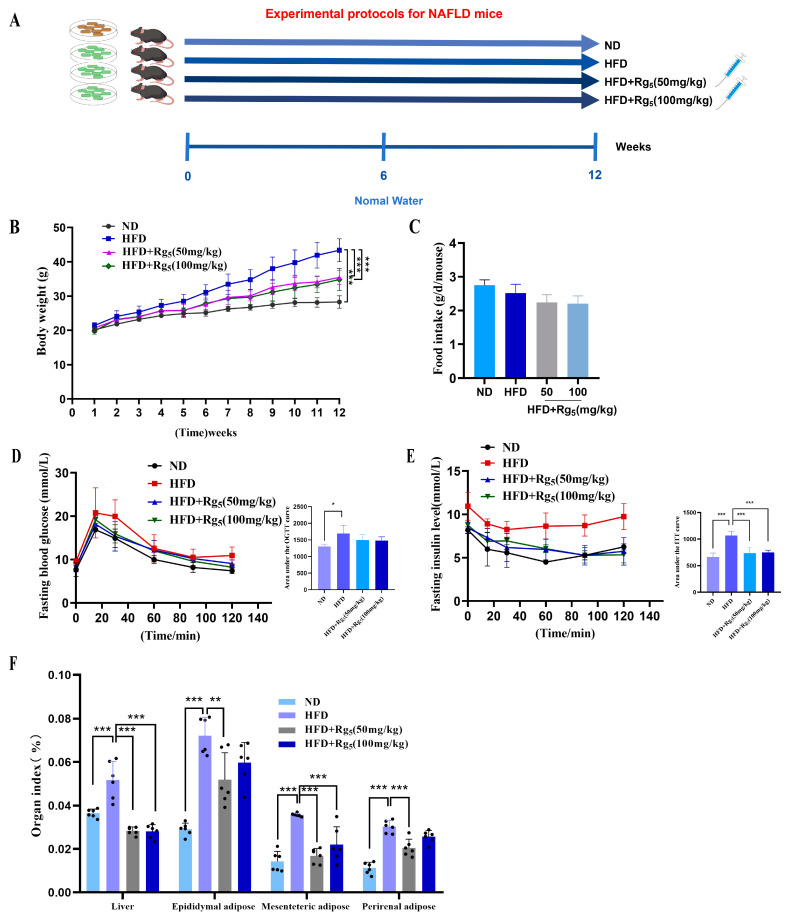
Experimental design protocol for NAFLD model, effects of Rg_5_ intervention on body weight, feed intake, and organ indexes in NAFLD mice. (**A**) Experimental design protocol for a mouse model of NAFLD where plain water is autoclaved distilled water. (**B**) Changes in body weight of mice. (**C**) The feed intake of mice. (**D**) Calculation of OGTT and corresponding AUC. (**E**) Calculation of ITT and corresponding AUC. (**F**) Liver, epididymal fat, mesenteric fat, and perirenal fat indexes. The black dots in the figure represent samples in each group. Data are expressed as mean ± SD; *n* = 6–10/group. *** *p* < 0.001, ** *p* < 0.05, * *p* < 0.05. *p* values were obtained via one-way ANOVA, multiple comparisons, and two-tailed Student’s *t*-test. The organ index was calculated using the following formula: organ index = [organ weight g/body weight g] × 100%.

To create a model of NAFLD, C57/BL6 mice were fed with an HFD for 12 weeks (Figure 1A). Throughout the experiment, all groups experienced an increase in body weight and food intake. As anticipated, following 12 weeks of intragastric administration, the mice on HFD had significantly higher body weights compared with ND (*p* < 0.05), while mice on HFD + Rg_5_ (50 mg/kg) and HFD + Rg_5_ (100 mg/kg) had significantly lower body weights after Rg_5_ treatment (Figure 1B). In addition, there were no significant differences in the food intake of the mice in each group (Figure 1C). Glucose and insulin levels were quantified in blood samples obtained during the OGTT and ITT in order to evaluate the metabolic status of glycemia in response to the treatment. As depicted in Figure 1D, blood glucose levels showed a significant increase within 30 min following oral glucose administration in all groups, subsequently declining thereafter. Compared to the ND group and the Rg_5_ ginsenoside group, the HFD group demonstrated markedly elevated blood glucose levels. The administration of Rg_5_ reversed the increase in the AUC of OGTT induced by the HFD (*p* < 0.001). Furthermore, the concentration of ITT in mice fed with HFD was higher than that in the ND group (*p* < 0.01), while supplementation with ginsenoside Rg_5_ decreased ITT levels (*p* < 0.001, Figure 1E). The results demonstrate that intervention with ginsenoside Rg_5_ significantly ameliorated glucose tolerance and insulin sensitivity in HFD-fed mice. The findings indicated that mice subjected to an HFD displayed more pronounced elevations in organ indices for hepatic, mesenteric fat, and perirenal fat in comparison to the group receiving a ND. However, this increase was significantly reversed via the intervention with Rg_5_ (Figure 1D).

### 3.2. Effects of Rg_5_ on the Phenotype and Protein Expression in NAFLD Mice

**Figure 2:**
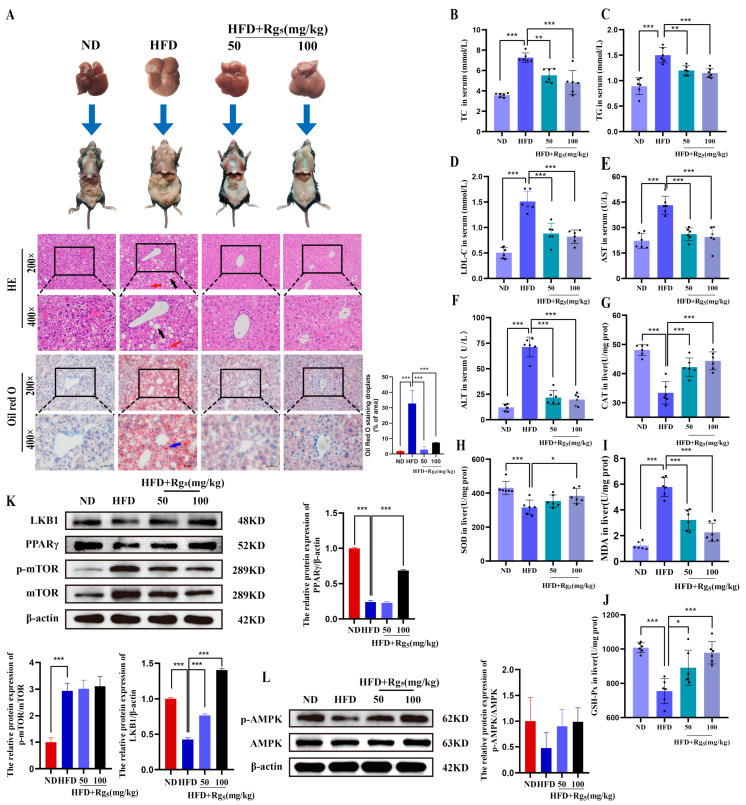
The histological examination of Rg_5_ intervention and its impact on lipids, liver damage, antioxidant activity, and hepatic protein expression levels in the liver of HFD mice were examined. Band density was used to quantify the amounts of proteins. (**A**) Histopathological analysis (H&E and Oil Red O staining) of liver tissue from mice in different groups at 200× and 400× magnification. Scale bar = 100 μm/50 μm. Black arrows represent fat vacuoles, red arrows indicate inflammatory infiltrates, and blue arrows denote lipid droplets. (**B**) TC in serum. (**C**) TG in serum. (**D**) LDL-C in serum. (**E**) AST in serum. (**F**) ALT in serum. (**G**) CAT in liver. (**D**) LDL-C in serum. (**H**) SOD in liver. (**I**) MDA in liver. (**J**) GSH-Px in liver. (**K**) Western blot analysis of p-AMPK, and AMPK in the liver. All data represent the results of three different experiments, mean ± SD (*n* = 3). (**L**) Western blot analysis of LKB1, p-mTOR, mTOR in the liver. All data represent the results of three different experiments, mean ± SD (*n* = 3). The black dots in the figure represent samples in each group.Data are expressed as mean ± SD (*n* = 6). *** *p* < 0.001, ** *p* < 0.01, * *p* < 0.05. *p* values were obtained via one-way ANOVA, multiple comparisons, and two-tailed Student’s *t*-test.

According to the H&E staining of liver tissues (Figure 2A), the liver lobules in the ND group appeared structurally organized and normal, with liver tissues of normal size and an orderly arrangement. In contrast, liver tissue from HFD mice exhibited more severe hepatocyte steatosis and inflammatory cell infiltration. However, hepatocellular steatosis improved after treatment with Rg_5_. The results of Oil Red O staining revealed a significantly greater red-stained area in the HFD group compared to the ND group, signifying a higher abundance of lipid droplets in the HFD group. This indicates that Rg_5_ intake significantly reduced lipid deposition in the liver and minimized HFD-induced liver injury. 

The lipid profiles of the four groups were measured at the end of the 12-week experiment. We found that the serum levels of TG, TC, LDL-C, AST, ALT, and MDA were significantly higher and the levels of SOD, CAT, and GSH-Px were substantially lower in the mice in the HFD group compared to the mice in the ND group. Furthermore, treatment with Rg_5_ reversed these effects and ameliorated the hepatic impairment and oxidative stress (Figure 2B–J).

Western blot results showed that, compared with the ND group, mice fed with HFD exhibited suppressed protein expression levels of LKB1 and p-AMPK, upregulated protein expression levels of p-mTOR, and Rg_5_ protein expression levels that tended to be similar to those of the normal group (Figure 2K,L). Additionally, these proteins are believed to promote lipolysis. The results of these studies indicate that Rg_5_ possesses the potential to enhance energy metabolism, facilitate lipid breakdown, and increase autophagic levels by activating LKB1/AMPK/mTOR, hence leading to the improvement of hepatic lipid accumulation in NAFLD.

### 3.3. Effects of Rg_5_ Intervention on the Intestinal Homeostasis of NAFLD Mice

**Figure 3:**
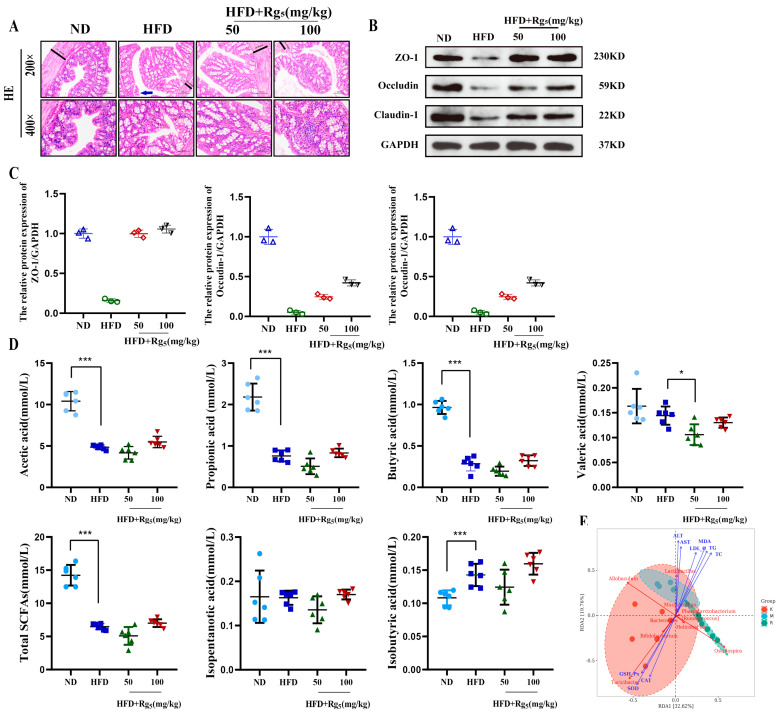
This study observed the effects of Rg_5_ on histopathological sections of mouse colon and epididymal fat as well as on the protein expression levels in the colon. Protein levels were quantified by measuring band density. (**A**) Histopathological analysis of colon of mice in different groups at 200× and 400× magnification. Scale bar = 100 μm/50 μm. The blue arrow indicates intestinal damage (**B**) Western blot protein images of ZO-1, Occludin, and Claudin-1 in the colon. (**C**) Western blot analysis of ZO-1, Occludin, and Claudin-1 in the colon. All data represent the results of three different experiments, mean ± SD (*n* = 3). (**D**) Effect of Rg_5_ intervention on acetic acid, propionic acid, butyric acid, valeric acid, isobutyric acid, isopentanoic acid, and total SCFA content. (**E**) RDA analysis of intestinal flora and basal indicators in mice. All data are expressed as mean ± SD, *n* = 6. *** *p* < 0.001, * *p* < 0.05. *p* values were obtained via one-way ANOVA, multiple comparisons, and two-tailed Student’s *t*-test.

The results reveal that within the HFD group, there is a decrease in the length of intestinal villi, accompanied by disorganized shedding and coinciding with the arrangement of intestinal epithelial cells. Additionally, the mucosal epithelial cells of the intestinal lining exhibit signs of atrophy, and the intrinsic muscle layer appears thinner. These observations collectively indicate significant impairment of the intestinal barrier. Importantly, it is worth noting that these changes undergo reversal in the Rg_5_ group (Figure 3A). To further explore the molecular mechanism of Rg_5_ treatment for intestinal injury protection, we performed Western blot experiments. To better observe their impact on intestinal mucosal integrity, we assessed the expression of epithelial tight-junction molecules (including ZO-1, Claudin-1, and Occludin) in the intestinal tissues of receptor mice. Next, we sought to determine whether Rg_5_ protected mice on an HFD from intestinal barrier damage. As expected, the expression of these proteins was significantly reduced in the HFD group compared with the ND group (*p* < 0.001), indicating disruption of the intestinal mucosal barrier. Conversely, the group treated with Rg_5_ via gavage exhibited a notable upregulation in the expression of these proteins, indicating the potential restoration of intestinal barrier function via Rg_5_ treatment (Figure 3B,C).

SCFAs are among the primary metabolites of the gut microbiota, playing crucial roles in host metabolism [21]. As reported in the literature, SCFAs derived from gut microbiota can serve as potent regulators of hepatic metabolic functions by acting as lipid precursors. Furthermore, they function as ligands for G-protein-coupled receptors (GPCRs), thereby modulating molecular signal transduction. This indicates that SCFAs play a more direct role in the gut–liver axis [22]. This segment of our study delved into the correlation between alterations in SCFAs and modifications of gut microbiota among mice afflicted with NAFLD. Our findings demonstrated notable alterations in the concentrations of six SCFAs within the HFD group when juxtaposed with the ND group. More specifically, a substantial decrease was observed in acetic acid, propionic acid, and butyric acid levels, while a significant elevation was noted in the content of isobutyric acid and the overall SCFAs content (Figure 3D). The administration of Rg_5_ exhibited a tendency to elevate the concentrations of these six SCFAs and the total SCFAs content in comparison to the HFD group, although this trend did not reach statistical significance. The findings propose that the administration of Rg_5_ could potentially disturb intestinal homeostasis by impacting the composition of the intestinal microbial community and its associated metabolism. However, following Rg_5_ treatment, there was a certain recovery effect, but it did not demonstrate significance. Following the analysis of our results, we posited that alterations in the contents of SCFAs correlate with various factors. The existing literature underscores feed as a crucial determinant shaping the composition of gut microorganisms. On the one hand, the diet serves to supply nutrients to the host; on the other hand, the feed acts as a substrate for the fermentation of mice gut microorganisms, thereby governing microbial growth, reproduction, and the concentration of their metabolites, SCFAs, consequently impacting gut health [23]. This is because the HFD used in our study was a commercially produced concentrate feed with a straightforward nutrient composition. Furthermore, the literature indicates that SCFAs can result from probiotic fermentation by specific gut bacteria, including *Bacteroides*, *Prevotella*, *Blautia*, and others, within the intestinal tract and colon. Rg_5_ may reduce the abundance of these bacteria and, as a result, decrease the content of acetic or propionic acid. The bacteria responsible for producing butyric acid and propionic acid are distinctly different, and this distinction is also a contributing factor to the lack of significant changes in SCFA levels between the two groups following intervention. This finding aligns with similar observations made in the literature [24]. We hypothesized that liver injury and disruptions in the intestinal environment caused by an HFD are linked to decreases in the concentration of total SCFAs and the bacteria responsible for their production. Previous studies have suggested that the production of butyrate is influenced by the expression and activity of specific enzymes, which can in turn be affected by the type of diet [25]. This is consistent with the pattern we observed in the overall structure of the gut microbiota. The findings indicate that Rg_5_ potentially exerts its anti-NAFLD activity via the modulation of gut metabolites, specifically SCFAs.

In the present study, we identify the connections between gut microorganisms and serological markers and emphasize their significance in regulating lipid metabolism. This information is crucial for examining the impact of modifications to gut flora on Rg_5_ in the treatment of NAFLD. Redundancy analysis (RDA) is a multivariate statistical method primarily used to explore the relationship between multivariate response data and one or more sets of explanatory variables [26]. The results showed that at the genus level, TC, TG, LDL-C, ALT, AST, and MDA were positively correlated with *Allobaulum* and *Lactobacillus*, while being negatively correlated with *Oscillospira*, *Bifidobacterium*, and *Turicibacter*. On the other hand, CAT, SOD, and GSH-Px were positively correlated with *Oscillospira*, *Turicibacter*, and *Bifidobacterium*, but negatively correlated with *Allobaulum* and *Lactobacillus* (Figure 3E). The results suggest that the changes in the abundance of these colonies are closely related to the changes in the structure of the intestinal flora in mice before and after Rg_5_ treatment.

### 3.4. Effects of Rg_5_ on Gut Microbiota Communities of NAFLD Mice

**Figure 4:**
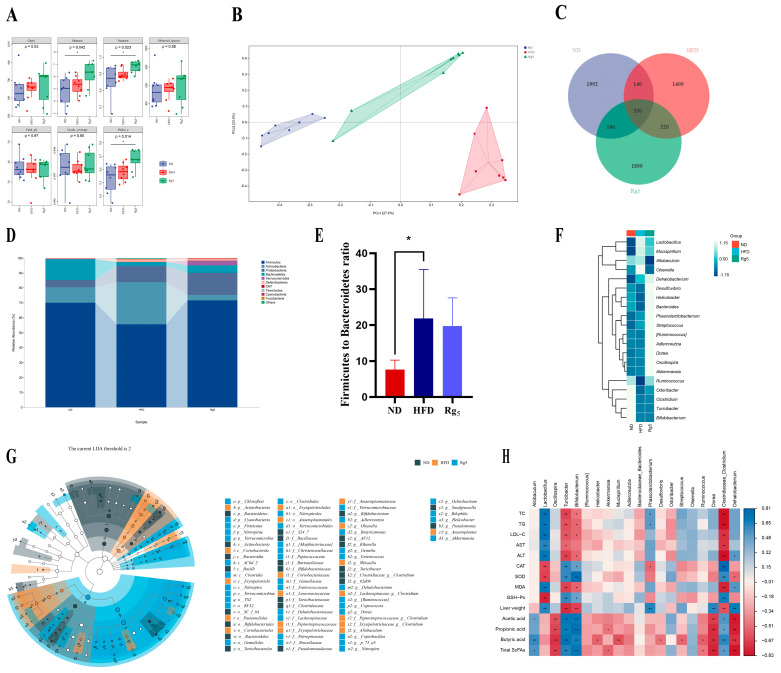
Effect of Rg_5_ intervention on the composition of the intestinal microbiota and screening of groups of characterized bacteria. (**A**) Chao1, Shannon, Simoson, observed species, Faith’s PD, Good’s coverage, and Pielou’s evenness indexes. (**B**) PCoA score plot of the gut microbiota among all groups, based on Bray–Curtis distances. (**C**) Wayne diagram of ASV/OUT. (**D**) Relative abundance analysis of the top 10 most abundant bacteria in the gut microbiota at the phylum level. (**E**) Ratio of *Firmicutes* to *Bacteoidetes*. (**F**) Heat map depicting species composition by subgroups. (**G**) Cladogram. (**H**) Heat map showing the Spearman correlation between glycolipid metabolism index, levels of SCFAs, and microorganisms at the genus levels in four groups of mice, respectively. Blue indicates high values, while red indicates low values. *n* = 6, significant correlations are indicated by * *p* < 0.05 and ** *p* < 0.01. All data are expressed as mean ± SD, *n* = 8. ** *p* < 0.01, * *p* < 0.05. *p* values were obtained via one-way ANOVA, multiple comparisons, and two-tailed Student’s *t*-test.

In order to further analyze the potential therapeutic effects of Rg_5_ on non-alcoholic fatty liver, we employed 16S rRNA sequencing to investigate the impact of Rg_5_ on the gut microbiota of NAFLD. The analysis of alpha diversity results revealed that, compared to the ND group, the Rg_5_ group exhibited increased species diversity and richness, while the HFD group did not show a significant decrease in species diversity and richness (Figure 4A). Principal coordinate analysis (PCoA) reveals a pronounced divergence in the composition of the intestinal microbiota across mice subjected to distinct dietary conditions (Figure 4B). This suggests that the gut microbial profiles of the mice underwent significant changes after supplementation with Rg_5_. The Venn diagram visually represents distinctions in the quantity of operational taxonomic units (OTUs), notably highlighting that the Rg_5_ group exhibits a significantly greater OTU count in comparison to the HFD group (Figure 4C).

Secondly, in terms of the species composition of the flora, the analysis at the phylum level analysis revealed that *Firmicutes*, *Bacteroides*, *Actinobacteria*, *Proteobacteria*, and *Verrucomicrobia* were among the most abundant bacteria in all groups of the top 10 phyla selected for this study (Figure 4D). In comparison to the HFD group, the Rg_5_ group exhibited a reduction in the *Firmicutes*/*Bacteroidetes* (*F/B*) ratio post-administration, signifying an amelioration in the gut microbiota environment of mice with NAFLD (Figure 4E). The abundance ratios of *Firmicutes* and *Bacteroidetes* exhibited significant elevations, serving as indicative markers for obesity [27]. This improvement helped to alleviate lipid metabolism disorders in the HFD mice to some extent.

In order to examine the differences in species composition between samples to an even greater extent, we conducted species composition analyses using heat maps of abundance data for the top 20 genera based on average abundance. The results of 16S rRNA gene sequencing analyses suggest that an HFD alters the composition of the gut flora. This finding is consistent with previous research. Compared to the ND group, the HFD intervention enriched the relative abundance of *Allobaculum*, *Olsenella*, and *Mucispirillum*, while also reducing the relative abundance of *Odoribacter* and *Bacteroide*. Under HFD conditions, supplementation with Rg_5_ increased the relative abundance of the genera *Akkermansia*, *Oscillospira, Phascolarctobacterium*, *Bacteroides*, and *Dehalobacterium* and decreased the relative abundance of the genera *Allobaculum* and *Olsenla* (Figure 4F). These findings serve as the basis of the theoretical groundwork for the prospective utilization of Rg_5_ as an innovative pharmaceutical, underpinning the formulation of strategies aimed at treating NAFLD by modulating the gut microbiota. In addition, our results showed that supplementation with Rg_5_ significantly upregulated the relative abundance of *Bacteroides*, a phylum of bacteria, and downregulated the relative abundance of *Allobaculum* and *Olsenella*. Gavage treatment with Rg_5_ significantly reduced the relative abundance of the harmful bacterium Helicobacter and promoted the growth of *Akkermansia* and *Oscillospira* (Figure 4F). Overall, the findings imply that the administration of Rg_5_ has the potential to ameliorate HFD-induced dysbiosis in intestinal microbiota, enhancing the abundance of certain key microorganisms, such as *Allobaculum*, *Akkermansia*, and *Coprococcus*. Utilizing the outcomes of LEfSe analysis, we successfully pinpointed the key microbial groups that experienced enrichment in the Rg_5_ group. The results showed that a total of 82 different bacterial species were screened in these three groups. There were 19, 23, and 40 significantly different genera in three groups, respectively (*p* < 0.05, LDA > 2) (Supplementary File Figure S1).

Subsequently, we conducted a Spearman association heat map analysis to examine the phenotypic correlation indices with the microorganisms of the intestinal flora (Figure 4H). As anticipated, in the phylum level analysis, the heat map results revealed that the *Firmicutes* and *Bacteroidetes* were negatively correlated with lipid related factors (TC, TG, and LDL-C) and liver damage indicators (AST, ALT), while being positively correlated with oxidative stress indicator (CAT, SOD, and GSH-Px) (Supplementary File Figure S2). Additionally, *Bacteroidetes* is also negatively correlated with acetic acid, propionic acid, butyric acid, and total SCFAs, as well as displaying a positive correlation with liver weight. At the genus level, the results align with those observed at the phylum level. From this perspective, the dysregulation of gut microbiota is directly associated with diminished inflammatory factors and a decrease in SCFA contents. Furthermore, this clarifies why the reduction in harmful bacteria and the increase in beneficial bacteria can efficiently regulate HFD-induced NAFLD in mice.

### 3.5. Effect of FMT on the Phenotype in NAFLD Mice

**Figure 5:**
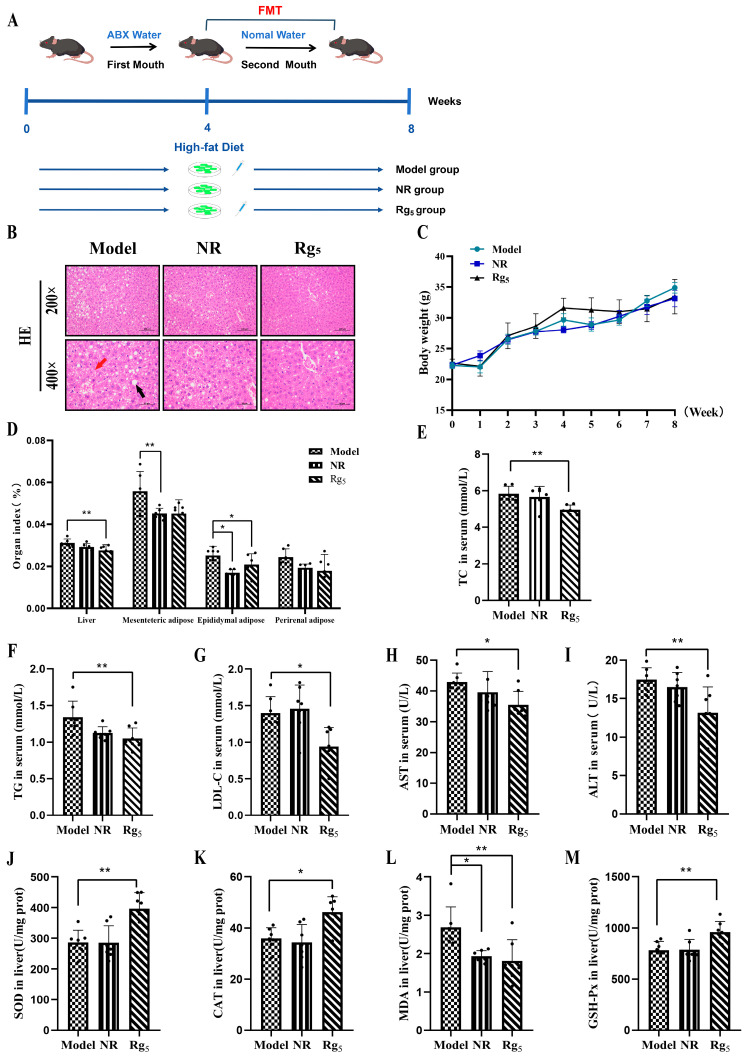
Design plan for fecal bacteria transplantation experiment and observation of phenotypes of each group of mice. (**A**) FMT experiment design proposal: normal water is distilled water after autoclaving, and ABX water is distilled water that has been mixed with four antibiotics. (**B**) H&E staining of liver tissue of mice in different groups at 200× and 400× magnification. Black arrows are fat vacuoles and red arrows are inflammatory infiltrates. (**C**) Changes in body weight of each group of mice during FMT experiments. (**D**) Organ index. (**E**) TC in serum. (**F**) TG in serum. (**G**) LDL-C in serum. (**H**) AST in serum. (**I**) ALT in serum. (**J**) SOD in liver. (**K**) CAT in liver. (**L**) MDA in liver. (**M**) GSH-Px in liver. The black dots in the figure represent samples in each group. Data are expressed as mean ± SD; *n* = 6/group; ** *p* < 0.01, * *p* < 0.05; *p* values were obtained via one-way ANOVA, multiple comparisons, and two-tailed Student’s *t*-test.

In order to validate the gut microbiota’s involvement in mediating host metabolic regulation and assess its effectiveness against NAFLD via FMT, we delved deeper into our investigation. The HFD group and the Rg_5_ group were administered, preparing fecal bacterial fluids via gavage. The donors for the model group were mice from the NAFLD model group, while the donors for the Rg_5_ group were mice from the HFD + Rg_5_ group (Figure 5A). According to the H&E staining of liver tissues (Figure 5B), we observed a decrease in vacuoles and inflammatory cell infiltration in hepatocytes, as well as reduced steatosis in the liver tissues of mice in the NR and Rg_5_ groups compared to the model group. After clearing the colony in the first month of antibiotic treatment, there was no significant difference in body weight among the three groups of mice, all of which grew steadily (note: during the two-month experimental period, all three groups were fed an HFD with 60% calories) (Figure 5C). The results showed that mice in the Rg_5_ group exhibited lower liver, mesenteric, and perirenal fat organ indices compared to the model group (Figure 5D). The lipid profiles of the three groups were measured at the end of the experiment in week 2. The serum levels of TG, TC, and LDL-C were significantly reduced in the Rg_5_ group of mice compared to the model group of mice. However, the effect was not significant in the NR group, although there was some degree of improvement (Figure 5E–G).

In addition, the serum levels of AST, ALT, and MDA were significantly reduced, and the levels of SOD, CAT, and GSH-Px showed significant increases in the Rg_5_ group of mice that were administered with fecal bacteria orally compared to those in the model group. However, the extent of recovery in the NR group was not very significant compared to the Rg_5_ group (Figure 5H–M). Overall, the obtained data indicate that FMT mitigates hepatic inflammation and oxidative stress in recipient mice, exhibiting a comparable effect to the treatment of donor mice with the Rg_5_ group.

### 3.6. Effect of FMT on Intestinal Bacterial Communities in NAFLD Mice

**Figure 6:**
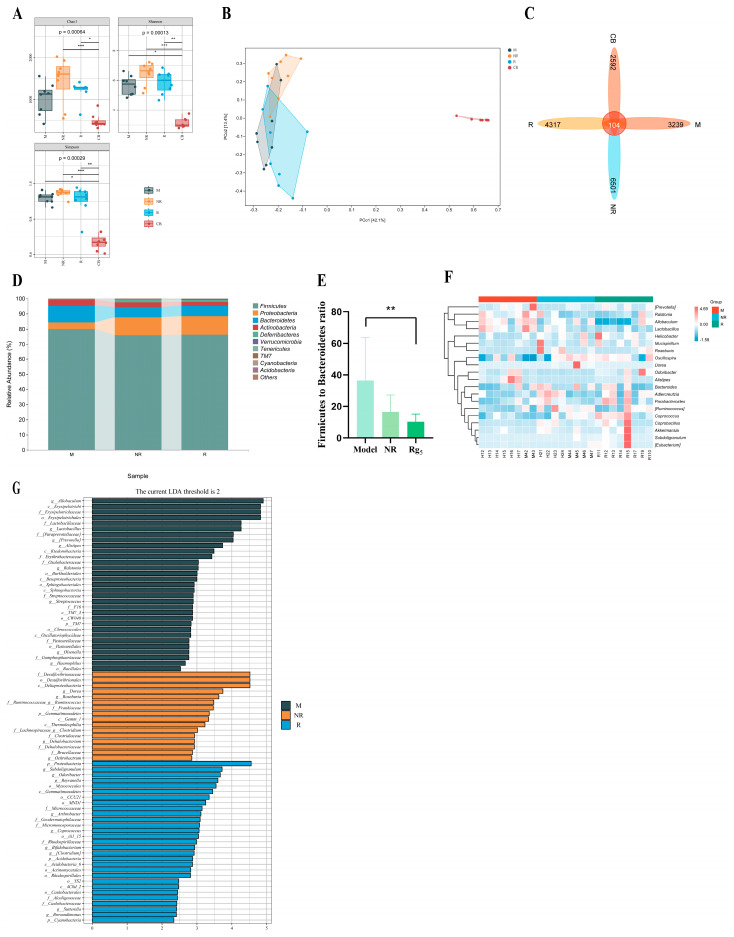
FMT from Rg_5_-treated donor mice reversed gut microbiota dysbiosis in HFD-fed recipient Mice. (**A**) Chao1 Shannon and Simpson indexes. (**B**) PCoA score plot of the gut microbiota among all groups, based on Bray-Curtis distances. (**C**) Wayne diagram of ASV/OTU. (**D**) Relative abundance analysis of the top 10 most abundant bacteria in the gut microbiota at the phylum level. (**E**) Ratio of *Firmicutes* to *Bacteoidetes*. (**F**) Heat map of species composition by sample and heat map of species composition by sub-groups. (**G**) LDA effect size analysis of characteristic genera of the gut microbiota (LEfSe). All data are expressed as mean ± SD, *n* = 8. *** *p* < 0.001, ** *p* < 0.01, * *p* < 0.05; *p* values were obtained using one-way ANOVA, multiple comparisons, and two-tailed Student’s *t*-test.

We conducted 16S rRNA gut flora sequencing on the feces of recipient mice to further investigate the differences in the fecal microbiota between NAFLD mice and FMT mice. Compared to model group treatment, treatment of the Rg_5_ group resulted in slight increases in the Chao1 index, Shannon index, and Simpson index, but the increases were not statistically significant. Furthermore, these three indices were significantly lower in the CB group compared to the other groups. This indicates that the antibiotic clearance of the flora during the initial premonitory period of the first month had a significant impact and should not be considered among the confounding factors affecting the experimental results (Figure 6A). According to the PCA score plot, there were notable structural variations in the gut microbiota between the Rg_5_ group and the model group (Figure 6B). The Venn diagram visually represents distinctions in the quantity of operational taxonomic units (OTUs) (Figure 6C). In addition, Proteobacteria increased at the portal level, while *Firmicutes*, *Bacteroides*, *Actinobacteria*, and *Deferribacteres* decreased in the Rg_5_ group compared to the model group with the NR sample (Figure 6D). It is also noteworthy that the *F/B* values were significantly lower following the gavage of Rg_5_ fecal bacteria treatment (Figure 6E). At the genus level, among the top 20 genera with significant differences, the relative abundance of certain potentially harmful genera such as *Allobaulum* and *Ralstonia,* as well as iterations with reduced harm such as *Roseburia*, *Oscillospira*, *Adlercreutzia*, and *Akkermansia*, were increased in the model group treated with gavage HFD fecal transplantation, Prarabacteroides, and the *Coprococcus* beneficial bacterial genera. The Rg_5_ group reinstated alterations in the relative abundance of these genera induced by the model group, enriching *Odoribacter*, *Subdoligranulum*, *Akkermansia*, *Coprococcus*, *Parabacteroides*, etc., and decreasing the relative abundance of *Allobaulum*, *Ralstonia*, and *Dore*. Furthermore, the NR group enriched the relative abundance of *Roseburia*, *Bacteroides*, and decreased the *Alistipes*, *Prevotella*, and *Odoribacter* relative abundance (Figure 6F). This discovery investigated the effects of supplementing with Rg_5_ in donor mice subjected to an HFD. We further identified specific colonies and biomarkers through LEfSe analysis to determine the dominant flora in each group (Figure 6G). We found that at the genus level, recipient mice in the model group were enriched in the genera *Allobaculum* and *Olsenella*. These genera were also dominant in donor mice in the HFD group. Additionally, *Coprococcus* was more prevalent in recipient mice in the Rg_5_ group than in donor mice. Intriguingly, *Akkermansia* predominated in donor mice in the Rg_5_ group, suggesting that these bacteria could potentially serve as biomarkers for identification purposes. Additionally, the gut microbiota of the recipient mice somewhat resembled that of the donor mice with which they were matched. These findings imply that FMT holds the potential to positively influence the enhancement of the gut microbiota environment, thus presenting it as a beneficial intervention against NAFLD.

## 4. Discussion

**Figure 7:**
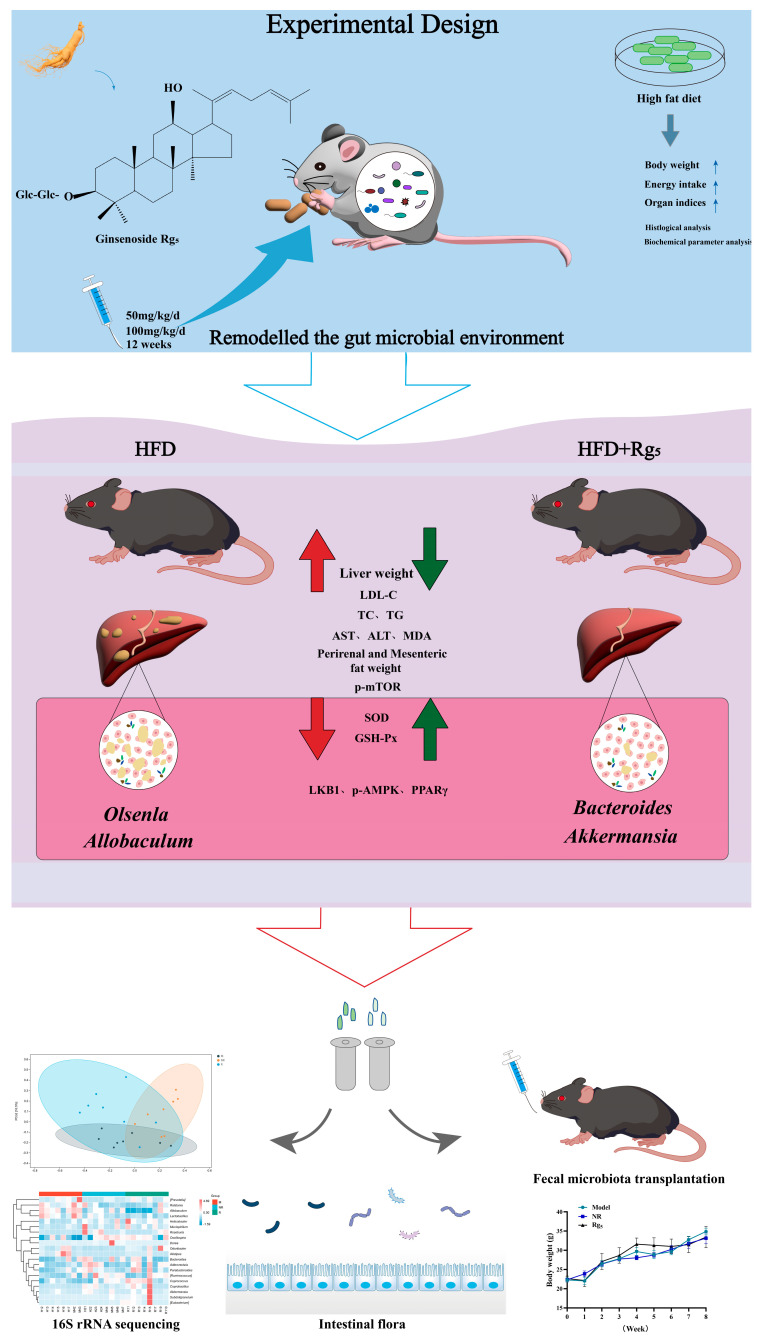
Exploring the therapeutic mechanism of ginsenoside Rg_5_ in NAFLD through fecal transplants targeting the intestinal microbiota. “↑” represents an increase in factor levels or protein expression, while “↓” represents a decrease in factor levels or protein expression.

NAFLD is an enduring, multifactorial, and intricate malady that is evolving into a burgeoning global health concern [28]. A plethora of studies has substantiated the anti-inflammatory, anti-obesity, anti-oxidative stress, and anti-NAFLD properties inherent in ginsenosides [29,30]. Although Rg_5_ has demonstrated a spectrum of physiological effects, recent investigations have predominantly emphasized its anticancer, anti-tumor, and anti-diabetic attributes, with potential implications as an anti-obesity medication [31,32,33]. Additionally, previous research has indicated that Rg_5_ possesses significant potential for treating NAFLD by repairing intestinal barrier function, alleviating inflammatory responses, inhibiting lipid accumulation, and preventing hepatocyte apoptosis [34,35]. Nevertheless, there is a paucity of definitive studies regarding the efficacy of Rg_5_ in treating NAFLD. Consequently, this current study was undertaken with the specific aim of elucidating the mechanistic foundations underlying the treatment of NAFLD by Rg_5_ (Figure 7). The primary objective of this research was to delineate the hepatoprotective benefits of Rg_5_ and unveil its underlying mechanisms, with a specific focus on its pivotal role in addressing NAFLD disease within the gut-microbiota-liver axis. In summary, our results revealed that treatment with Rg_5_ can not only markedly diminish the ultimate body weight of mice afflicted with NAFLD but can also attenuate hepatic fat degeneration and mitigate the infiltration of inflammation. Additionally, it exerts a discernible effect in terms of reducing blood lipid levels while ameliorating oxidative stress. Subsequently, we undertook comprehensive investigations into its intricate molecular mechanisms. These included the activation of the liver LKB1/AMPK/mTOR pathway, the observed increase in expression of intestinal tight junction proteins, and alterations in the overall composition of the intestinal microbiota, characterized by an augmentation in beneficial bacterial genera and a reduction in detrimental bacteria. Collectively, these favorable alterations exert suppressive effects on NAFLD induced by hyperlipidemia. As is widely acknowledged, AMP-activated protein kinase (AMPK) stands out as a crucial regulator in lipid metabolism. In situations of energy deficiency, the AMPK pathway undergoes phosphorylation and activation, facilitated by the upstream liver kinase B1 (LKB1), which plays a role assisting in energy metabolism [36]. mTOR functions as a downstream target of LKB1/AMPK, engaging in diverse biological processes, including protein synthesis and energy metabolism. Furthermore, its participation in the autophagic process facilitates the degradation of cellular components, playing a pivotal role in lipid generation and metabolism [37]. The primary function of autophagy is to facilitate the degradation of cellular organelles and various cellular constituents, which assumes a crucial role in both the generation and metabolism of lipids [38]. Surprisingly, research reports that HFD mice display relatively high mTORC1 signaling, which may be related to the increase in pro-inflammatory cytokine levels, consistent with our research findings [39]. In our research, we observed the activation of the LKB1/AMPK/mTOR pathway, which suppressed HFD-induced NAFLD by enhancing energy metabolism and modulating autophagy. Concurrently, Rg_5_ upregulated the expression of tight-junction proteins ZO-1, Occludin, and Claudin-1, thereby restoring intestinal damage and ensuring the integrity of the intestinal barrier. These findings suggest that the LKB1/AMPK/mTOR signaling pathway possesses pivotal role in suppressing the progression of NAFLD, particularly in facilitating lipid breakdown and augmenting energy metabolism. Moreover, Rg_5_ exhibited a notable reduction in liver lipid accumulation and oxidative stress in NAFLD mice, surpassing the effects observed in the HFD group. This also mitigated symptoms associated with lipid metabolism disorders. Research indicates that alterations in the gut microbiota environment play crucial roles in the onset and progression of NAFLD. Both beneficial and detrimental gut metabolites are directly transported to the liver via the portal vein, contributing to a range of biological processes, encompassing energy production, detoxification, and synthesis [40,41,42,43]. In this study, we scrutinized the regulatory influence of Rg_5_ intervention on the gastrointestinal flora of NAFLD mice. Ultimately, we observed that Rg_5_ reversed alterations in the flora, modified overall composition, and augmented the abundance of various beneficial genera, including *Akkermansia*, *Oscillospira*, and *Bacteroide*.

Furthermore, in order to ascertain whether the favorable effects of Rg_5_ were contingent upon the gut microbiota, we conducted FMT analyses. The findings indicated that the anti-NAFLD effects of Rg_5_ were transferrable via fecal transplantation. Notably, there was a close association between the gut microbiota and the host. This association plays a pivotal role in the pathological advancement of NAFLD by impacting energy storage and lipid processing [44]. Alterations in the *F/B* ratio exhibited a significant correlation with changes in body weight, with an elevated *F*/*B* ratio being linked to obesity [45]. Our data revealed that Rg_5_ treatment significantly inhibited the elevation of the *Bacteroides*. The *F/B* ratio was induced by an HFD, indicating a potential mechanism through which Rg_5_ may counteract the development of NAFLD. Hence, comprehending the mechanisms underlying these processes, including alterations in specific genera (*Olsenella*, *Akkermansia*, *Allobaculum*, *Helicobacter*, *Adlercreutzia*, *Dorea*, etc.), may be pivotal in the treatment or prevention of NAFLD. Identified as a prospective candidate for the next generation of probiotics, *Oscillospira* assumes a crucial role in metabolism, harboring substantial potential for fostering weight loss, reducing cholesterol levels, and ameliorating metabolic syndrome [46]. Intriguingly, the administration of Rg_5_ markedly elevated the abundance of *Oscillospira.* Presently, accumulating evidence suggests that the genus *Akkermansia* functions as a probiotic in the gastrointestinal tract. Furthermore, a negative correlation exists between the abundance of *Akkermansia* and obesity [47,48]. These results align with our observation that the abundance of the genus *Akkermansia* was markedly diminished within our HFD group. Nevertheless, this reduction was effectively reversed via Rg_5_ treatment in our investigation. Remarkably, *Allobaculum*, a member of the phylum *Firmicutes*, acts as a producer of lactate and butyrate. Research evidence suggests that its abundance exhibits a negative correlation with inflammation and obesity. However, our data present less consistency as they reveal predominant enrichment in the HFD group. Yet, the existing literature proposes a similar positive correlation between the expression of the key regulator of lipid metabolism and the abundance of *Allobaculum* [49]. The reason for this difference may be attributed to variations in the composition of the mouse gut microbiota. In addition, another crucial aspect concerning the relationship between NAFLD and the gut microbiota is the production of SCFAs. Diverse studies have substantiated the role of SCFAs as potential mediators of the gut microbiota, exerting influence on hepatic metabolic functions and modulating the progression of NAFLD. Organisms including *Bacteroides*, *Roseburia*, *Akkermansia*, and others have been recognized as producers of SCFAs [50]. Through fermentation, these bacteria synthesize SCFAs such as butyrate, propionate, and acetate, which serve as abundant energy sources for their hosts [51]. As an emerging drug, Rg_5_ has excellent potential for the prevention and treatment of metabolic disorders such as NAFLD, obesity, and diabetes mellitus [52]. In addition, targeting the gut microbiota may be a new preventive or therapeutic approach to the enhancement of NAFLD [53]. It has been shown that the human gut microbiota can be transferred to mice while maintaining its structure and diversity, and fecal transplants are currently being used to treat diseases caused by *Clostridium* difficile infection [54]. Based on this, we can anticipate the use of human fecal transplants for the treatment of more significant metabolic disorders like NAFLD in the future.

## 5. Conclusions

This study investigated the effects of Rg_5_ on the phenotype of NAFLD induced by HFD in mice, including its key role in inhibiting the development of non-alcoholic fatty liver through the LKB1/AMPK/mTOR signaling pathway and the gut–microbiota–liver axis. The results indicated that treatment with Rg_5_ in NAFLD mice reduced liver fat degeneration and colonic inflammatory damage, notably improving biochemical indicators. The intervention group treated with Rg_5_ also exhibited decreased expression of oxidative stress markers. This indicated that the Rg_5_ intervention effectively improved oxidative stress and liver damage in mice with NAFLD. In summary, our investigation illuminates the activation of the LKB1/AMPK/mTOR axis by Rg_5_, implying its engagement in the regulation of metabolic dysfunction. This suggests that Rg_5_ may alleviate the mechanisms contributing to NAFLD through this particular pathway. According to the 16S rRNA sequencing results, the intervention with Rg_5_ significantly increased the abundance of beneficial bacteria, such as *Bacteroides* and *Akkermansia*. FMT also produced similar results. Furthermore, Rg_5_ also improved intestinal barrier damage by increasing the protein expression levels of ZO-1, Occludin, and Claudin-1, maintaining the integrity of the intestinal barrier. This also resulted in elevated levels of SCFAs, indicating that Rg_5_ supplementation can promote a beneficial gut microbiota environment. This finding may also pave the way for a new research direction for the prevention and treatment of NAFLD. The results indicated that treatment with Rg_5_ in NAFLD mice reduced liver fat degeneration and colonic inflammatory damage, considerably improving biochemical indicators. The intervention group treated with Rg_5_ also exhibited decreased expression of oxidative stress markers. This indicates that the Rg_5_ intervention effectively improved oxidative stress and liver damage in mice with NAFLD. In summary, our investigation illuminates the activation of the LKB1/AMPK/mTOR axis by Rg_5_, implying its engagement in the regulation of metabolic dysfunction. This suggests that Rg_5_ may alleviate the mechanisms contributing to NAFLD via this particular pathway. According to the 16S rRNA sequencing results, the intervention with Rg_5_ significantly increased the abundance of beneficial bacteria, such as *Bacteroides* and *Akkermansia*. FMT also produced similar results. In terms of the mechanism by which Rg_5_ treats NAFLD by modulating the gut microbiota, while the involvement through the gut-liver axis may not seem directly straightforward in terms of transmission, its therapeutic effect on liver diseases remains critically important. Furthermore, Rg_5_ limited intestinal barrier damage by increasing the protein expression levels of ZO-1, Occludin, and Claudin-1, maintaining the integrity of the intestinal barrier. This also resulted in elevated levels of SCFAs, indicating that Rg_5_ supplementation can promote a beneficial gut microbiota environment. This finding also paves the way for a new research direction in preventing and treating NAFLD.

## Data Availability

The data for this study is included in both the article and Supplementary Materials.

## Supplementary Materials

The following supporting information can be downloaded at: https://www.mdpi.com/article/10.3390/nu16060842/s1, Table S1. Low-fat mouse chow with 10% kcal from fat. Table S2. High-fat mouse chow with 60% kcal from fat. Figure S1. LDA effect size analysis effect size analysis of characteristic genera of the gut microbiota (LEfSe). Figure S2. Heat map showing the Spearman correlation between glycolipid metabolism index, levels of SCFAs, and microorganisms at the phylum and genus levels in four groups of mice, respectively. Blue in-dicates high values, while red indicates low values. n=6, significant correlations are indicated by * *p* < 0.05 and ** *p* < 0.01. References [55,56,57,58,59,60,61,62,63,64,65] are cited in the supplementary materials.

## References

[1] Powell E.E., Wong V.W.S., Rinella M. (2021). Non-alcoholic fatty liver disease. Lancet.

[2] Friedman S.L., Neuschwander-Tetri B.A., Rinella M., Sanyal A.J. (2018). Mechanisms of NAFLD development and therapeutic strategies. Nat. Med..

[3] Youssefian L., Vahidnezhad H., Saeidian A.H., Pajouhanfar S., Sotoudeh S., Mansouri P., Amirkashani D., Zeinali S., Levine M.A., Peris K. (2019). Inherited non-alcoholic fatty liver disease and dyslipidemia due to monoallelic ABHD5 mutations. J. Hepatol..

[4] Aron-Wisnewsky J., Vigliotti C., Witjes J., Le P., Holleboom A.G., Verheij J., Nieuwdorp M., Clément K. (2020). Gut microbiota and human NAFLD: Disentangling microbial signatures from metabolic disorders. Nat. Rev..

[5] Liu J., Wu A., Cai J., She Z.-G., Li H. (2022). The contribution of the gut-liver axis to the immune signaling pathway of NAFLD. Front. Immunol..

[6] Makki K., Deehan E.C., Walter J., Bäckhed F. (2018). The Impact of Dietary Fiber on Gut Microbiota in Host Health and Disease. Cell Host Microbe.

[7] Roselli M., Natella F., Zinno P., Guantario B., Canali R., Schifano E., De Angelis M., Nikoloudaki O., Gobbetti M., Perozzi G. (2021). Colonization ability and impact on hu-man gut microbiota of foodborne microbes from traditional or probiotic-added fermented foods: A systematic review. Front. Nutr..

[8] Vijay A., Valdes A.M. (2022). Role of the gut microbiome in chronic diseases: A narrative review. Eur. J. Clin. Nutr..

[9] Su Y., Li J., Wu L., Kuang H. (2021). Polysaccharides from TCM herbs exhibit potent anti-obesity effect by mediating the community structure of gut microbiota. Pharmazie.

[10] Wu Z., Tian E., Chen Y., Dong Z., Peng Q. (2023). Gut microbiota and its roles in the pathogenesis and therapy of en-do-crine system diseases. Microbiol. Res..

[11] Blaak E.E., Canfora E.E., Theis S., Frost G., Groen A.K., Mithieux G., Nauta A., Scott K., Stahl B., Van Harsselaar J. (2020). Short chain fatty acids in human gut and metabolic health. Benef. Microbes.

[12] Chen C., Lv Q., Li Y., Jin Y.H. (2021). The Anti-Tumor Effect and Underlying Apoptotic Mechanism of Ginsenoside Rk1 and Rg5 in Human Liver Cancer Cells. Molecules.

[13] Wang J., Zhang X., Yang X., Yu H., Bu M., Fu J., Zhang Z., Xu H., Hu J., Lu J. (2023). Revitalizing myocarditis treatment through gut microbiota modulation: Unveiling a promising therapeutic avenue. Front. Cell. Infect. Microbiol..

[14] Gao P., Zheng M., Lu H., Lu S. (2023). The progressive utilization of ponkan peel residue for regulating human gut microbiota through sequential extraction and modification of its dietary fibers. Foods.

[15] Qian J., Lu J., Cheng S., Zou X., Tao Q., Wang M., Wang N., Zheng L., Liao W., Li Y. (2023). Periodontitis salivary microbiota exacerbates colitis-induced anxiety-like behavior via gut microbiota. Npj Biofilms Microbiomes.

[16] Bai Z., Huang X., Wu G., Ye H., Huang W., Nie Q., Chen H., Yin J., Chen Y., Nie S. (2023). Polysaccharides from red kidney bean alleviating hyperglycemia and hyperlipidemia in type 2 diabetic rats via gut microbiota and lipid metabolic modulation. Food Chem..

[17] Bolyen E., Rideout J.R., Dillon M.R., Bokulich N.A., Abnet C.C., Al-Ghalith G.A., Alexander H., Alm E.J., Arumugam M., Asnicar F. (2019). Reproducible, in-teractive, scalable and extensible microbiome data science using QIIME 2. Nat. Biotechnol..

[18] Bokulich N.A., Kaehler B.D., Rideout J.R., Dillon M., Bolyen E., Knight R., Huttley G.A., Gregory Caporaso J. (2018). Optimizing taxonomic classification of marker-gene amplicon sequences with QIIME 2′s q2-feature-classifier plugin. Microbiome.

[19] Ren D.D., Chen K.C., Li S.S., Zhang Y.T., Li Z.M., Liu S., Sun Y.S. (2023). Panax quinquefolius polysaccharides ameliorate ulcerative colitis in mice induced by dextran sulfate sodium. Front. Immunol..

[20] Zheng Z., Lyu W., Ren Y., Li X., Zhao S., Yang H., Xiao Y. (2021). Allobaculum involves in the modulation of intestinal ANGPTLT4 expression in mice treated by high-fat diet. Front. Nutr..

[21] Zhang D., Jian Y.P., Zhang Y.N., Li Y., Gu L.T., Sun H.H., Liu M.D., Zhou H.L., Wang Y.S., Xu Z.X. (2023). Short-chain fatty acids in diseases. Cell Commun. Signal. CCS.

[22] Han H., Jiang Y., Wang M., Melaku M., Liu L., Zhao Y., Everaert N., Yi B., Zhang H. (2023). Intestinal dysbiosis in nonalcoholic fatty liver disease (NAFLD): Focusing on the gut-liver axis. Crit. Rev. Food Sci. Nutr..

[23] Everard A., Lazarevic V., Gaïa N., Johansson M., Ståhlman M., Backhed F., Delzenne N.M., Schrenzel J., François P., Cani P.D. (2014). Microbiome of prebiotic-treated mice reveals novel targets involved in host response during obesity. ISME J..

[24] Bao T., He F., Zhang X., Zhu L., Wang Z., Lu H., Wang T., Li Y., Yang S., Wang H. (2020). Inulin exerts beneficial effects on non-alcoholic fatty liver disease via modulating gut microbiome and suppressing the lipopolysaccha-ride-toll-like receptor 4-Mψ-nuclear factor-κB-nod-Like receptor protein 3 pathway via gut-liver axis in mice. Front. Pharmacol..

[25] Yu M., Li Z., Chen W., Rong T., Wang G., Ma X. (2019). Microbiome-metabolomics analysis investigating the impacts of dietary starch types on the composition and metabolism of colonic microbiota in finishing pigs. Front. Microbiol..

[26] Csala A., Hof M.H., Zwinderman A.H. (2019). Multiset sparse redundancy analysis for high-dimensional omics data. Biom. J..

[27] Xie Z., Du J., Gan M., Zhou C., Li M., Liu C., Wang M., Chen L., Zhao Y., Wang Y. (2023). Short-term dietary choline supplementation alters the gut microbiota and liver metabolism of finishing pigs. Front. Microbiol..

[28] Younossi Z.M., Koenig A.B., Abdelatif D., Fazel Y., Henry L., Wymer M. (2016). Global epidemiology of nonalcoholic fatty liver disease-Meta-analytic assessment of prevalence, incidence, and outcomes. Hepatology.

[29] Jin W., Li C., Yang S., Song S., Hou W., Song Y., Du Q. (2023). Hypolipidemic effect and molecular mechanism of ginsenosides: A review based on oxidative stress. Front. Pharmacol..

[30] Liang W., Zhou K., Jian P., Chang Z., Zhang Q., Liu Y., Xiao S., Zhang L. (2021). Ginsenosides improve nonalcoholic fatty liver disease via integrated regulation of gut microbiota, inflammation and energy homeostasis. Front. Pharmacol..

[31] Zhu Y., Zhu C., Yang H., Deng J., Fan D. (2020). Protective effect of ginsenoside Rg_5_ against kidney injury via inhibition of NLRP3 inflammasome activation and the MAPK signaling pathway in high-fat diet/streptozotocin-induced diabetic mice. Pharmacol. Res..

[32] Liu M.Y., Liu F., Gao Y.L., Yin J.N., Yan W.Q., Liu J.G., Li H.J. (2021). Pharmacological activities of ginsenoside Rg_5_ (Review). Exp. Ther. Med..

[33] Xiao N., Lou M.D., Lu Y.T., Yang L.L., Liu Q., Liu B., Qi L.W., Li P. (2017). Ginsenoside Rg_5_ attenuates hepatic glucagon response via suppression of succinate-associated HIF-1α induction in HFD-fed mice. Diabetologia.

[34] Wei Y., Yang H., Zhu C., Deng J., Fan D. (2020). Hypoglycemic Effect of Ginsenoside Rg5 Mediated Partly by Modulating Gut Microbiota Dysbiosis in Diabetic db/db Mice. J. Agric. Food Chem..

[35] Li N., Zhu C., Fu R., Ma X., Duan Z., Fan D. (2024). Ginsenoside Rg5 inhibits lipid accumulation and hepatocyte apoptosis via the Notch1 signaling pathway in NASH mice. Phytomedicine.

[36] Garcia D., Shaw R.J. (2017). AMPK: Mechanisms of cellular energy sensing and restoration of metabolic balance. Mol. Cell.

[37] Xiong Y., Xu Z., Wang Y., Kuang S., Shan T. (2018). Adipocyte-specific DKO of Lkb1 and mTOR protects mice against HFD-induced obesity, but results in insulin resistance. J. Lipid Res..

[38] Panwar V., Singh A., Bhatt M., Tonk R.K., Azizov S., Raza A.S., Sengupta S., Kumar D., Garg M. (2023). Multifaceted role of mTOR (mammalian target of rapamycin) signaling pathway in human health and disease. Signal Transduct. Target. Ther..

[39] Saxton R.A., Sabatini D.M. (2017). mTOR Signaling in Growth, Metabolism, and Disease. Cell.

[40] Zhu W., Zhou Y., Tsao R., Dong H., Zhang H. (2022). Amelioratory effect of resistant starch on non-alcoholic fatty liver disease via the gut-liver axis. Front. Nutr..

[41] Zhang Q., Xing W., Wang Q., Tang Z., Wang Y., Gao W. (2022). Gut microbiota-mitochondrial inter-talk in nonalcoholic fatty liver disease. Front. Nutr..

[42] Hrncir T., Hrncirova L., Kverka M., Hromadka R., Machova V., Trckova E., Kostovcikova K., Kralickova P., Krejsek J., Tlaskalova-Hogenova H. (2021). Gut Microbiota and NAFLD: Pathogenetic Mechanisms, Microbiota Signatures, and Therapeutic Interventions. Microorganisms.

[43] Plaza-Díaz J., Solis-Urra P., Aragón-Vela J., Rodríguez-Rodríguez F., Olivares-Arancibia J., Álvarez-Mercado A.I. (2021). Insights into the Impact of Microbiota in the Treatment of NAFLD/NASH and Its Potential as a Biomarker for Prognosis and Diagnosis. Biomedicines.

[44] Rodríguez-Daza M.C., Pulido-Mateos E.C., Lupien-Meilleur J., Guyonnet D., Desjardins Y., Roy D. (2021). Polyphenolmediated gut microbiota modulation: Toward prebiotics and further. Front. Nutr..

[45] Yin J., Li Y., Han H., Ma J., Liu G., Wu X., Huang X., Fang R., Baba K., Bin P. (2020). Administration of exogenous melatonin improves the diurnal rhythms of the gut microbiota in mice fed a high-fat diet. mSystems.

[46] Yang J., Li Y., Wen Z., Liu W., Meng L., Huang H. (2021). Oscillospira-A candidate for the next-generation probiotics. Gut Microbes.

[47] Carvalho B.M., Guadagnini D., Tsukumo D.M.L., Schenka A.A., Latuf-Filho P., Vassallo J., Dias J.C., Kubota L.T., Carvalheira J.B.C., Saad M.J.A. (2012). Modulation of gut microbiota by antibiotics improves insulin signalling in high-fat fed mice. Diabetologia.

[48] Depommier C., Everard A., Druart C., Plovier H., Van Hul M., Vieira-Silva S., Falony G., Raes J., Maiter D., Delzenne N.M. (2019). Supplementation with Ak-ker-mansia muciniphila in overweight and obese human volunteers: A proof-of-concept exploratory study. Nat. Med..

[49] van Muijlwijk G.H., van Mierlo G., Jansen P.W.T.C., Vermeulen M., Bleumink-Pluym N.M.C., Palm N.W., van Putten J.P.M., de Zoete M.R. (2021). Identification of Allobaculum mucolyticum as a novel human intestinal mucin degrader. Gut Microbes.

[50] Hong Y., Sheng L., Zhong J., Tao X., Zhu W., Ma J., Yan J., Zhao A., Zheng X., Wu G. (2021). Desulfovibrio vulgaris, a potent acetic acid-producing bacterium, attenuates nonalcoholic fatty liver disease in mice. Gut Microbes.

[51] Zikou E., Dovrolis N., Dimosthenopoulos C., Gazouli M., Makrilakis K. (2023). The effect of probiotic supplements on metabolic parameters of people with type 2 diabetes in greece-a randomized, double-blind, placebo-controlled study. Nutrients.

[52] Cotillard A., Kennedy S.P., Kong L.C., Prifti E., Pons N., Le Chatelier E., Almeida M., Quinquis B., Levenez F., Galleron N. (2013). Dietary intervention impact on gut microbial gene richness. Nature.

[53] Xue L., Deng Z., Luo W., He X., Chen Y. (2022). Effect of fecal microbiota transplantation on non-alcoholic fatty liver disease: A randomized clinical trial. Front. Cell. Infect. Microbiol..

[54] Tariq R., Syed T., Yadav D., Prokop L.J., Singh S., Loftus E.V., Pardi D.S., Khanna S. (2023). Outcomes of fecal microbiota transplantation for C. difficile infection in inflammatory bowel disease: A systematic review and meta-analysis. J. Clin. Gastroenterol..

[55] Coia H., Ma N., Hou Y., Dyba M.D., Fu Y., Cruz M.I., Benitez C., Graham G.T., McCutcheon J.N., Zheng Y.L. (2018). Prevention of Lipid Peroxidation-derived Cyclic DNA Adduct and Mutation in High Fat Diet-induced Hepatocarcinogenesis by Theaphenon E. Cancer Prev. Res..

[56] Guo J., Pereira T.J., Dalvi P., Yeung L.S.N., Swain N., Breen D.M., Lam L., Dolinsky V.W., Giacca A. (2016). High-dose metformin (420 mg/kg daily p.o.) increases insulin sensitivity but does not affect neointimal thickness in the rat carotid balloon injury model of restenosis. Metabolism.

[57] Norris G.H., Porter C.M., Jiang C., Millar C.L., Blesso C.N. (2017). Dietary sphingomyelin attenuates hepatic steatosis and adipose tissue inflammation in high-fat-diet-induced obese mice. J. Nutr. Biochem..

[58] Tordoff M.G., Aleman T.R., Murphy M.C. (2012). No effects of monosodium glutamate consumption on the body weight or composition of adult rats and mice. Physiol. Behav..

[59] Lee Y.J., Ko E.H., Kim J.E., Kim E., Lee H., Choi H., Yu J.H., Kim H.J., Seong J.K., Kim K.S. (2012). Nuclear receptor PPARγ-regulated monoacylglycerol O-acyltransferase 1 (MGAT1) expression is responsible for the lipid accumulation in diet-induced hepatic steatosis. Proc. Natl. Acad. Sci. USA.

[60] Perry R.J., Resch J.M., Douglass A.M., Madara J.C., Rabin-Court A., Kucukdereli H., Wu C., Song J.D., Lowell B.B., Shulman G.I. (2020). Leptin’s hunger-suppressing effects are mediated by the hypothalamic–pituitary–adrenocortical axis in rodents. ESPE Yearb. Paediatr. Endocrinol..

[61] Miranda C.L., Johnson L.A., de Montgolfier O., Elias V.D., Ullrich L.S., Hay J.J., Paraiso I.L., Choi J., Reed R.L., Revel J.S. (2018). Non-estrogenic Xanthohumol Derivatives Mitigate Insulin Resistance and Cognitive Impairment in High-Fat Diet-induced Obese Mice. Sci. Rep..

[62] Mitchell S.J., Bernier M., Aon M.A., Cortassa S., Kim E.Y., Fang E.F., Palacios H.H., Ali A., Navas-Enamorado I., Di Francesco A. (2018). Nicotinamide Improves Aspects of Healthspan, but Not Lifespan, in Mice. Cell Metab..

[63] Kopec A.K., Abrahams S.R., Thornton S., Palumbo J.S., Mullins E.S., Divanovic S., Weiler H., Owens A.P., Mackman N., Goss A. (2017). Thrombin promotes diet-induced obesity through fibrin-driven inflammation. J. Clin. Investig..

[64] Yang J.W., Kim H.S., Im J.H., Kim J.W., Jun D.W., Lim S.C., Lee K., Choi J.M., Kim S.K., Kang K.W. (2016). GPR119: A promising target for nonalcoholic fatty liver disease. FASEB J..

[65] Miyao M., Kotani H., Ishida T., Kawai C., Manabe S., Abiru H., Tamaki K. (2015). Pivotal role of liver sinusoidal endothelial cells in NAFLD/NASH progression. Mod. Pathol..

