# Supplementary material for: Ginsenoside Rg5 Activates the LKB1/AMPK/mTOR Signaling Pathway and Modifies the Gut Microbiota to Alleviate Nonalcoholic Fatty Liver Disease Induced by a High-Fat Diet
Source: Nutrients. 2024 Mar 15;16(6):842. doi: 10.3390/nu16060842 (PMC10974897; doi:10.3390/nu16060842)
Supplement: Supplementary file 1 [file nutrients-16-00842-s001.zip › nutrients-2870804-supplementary Tables.pdf]

nutrients

MDPI

Table S1. Low-fat mouse chow with 10% kcal from fat.

	gm%	Kcal%
Protein	19.2	20
Carbohydrate	67.3	70
Fat	4.3	10
Kcal/gm	3.86	

Ingredient	Caloric Value Per Unit (kcal/g)	Weight (gram)	Calories (kcal)
Caisein	4	200	800
L-Cystine	4	3	12
Sucrose	4	68.8	275.2
Dyetrose	4	125	500
Cornstarch	4	506.2	2024.8
Lard	9	20	180
Soybean Oil	9	25	225
Cellulose	0	50	0
Mineral Mix#210088	1.6	10	16
Calcium Carbonate	0	5.5	0
Dicalcium Phosphate	0	13	0
Potassium Citrate H20	0	16.5	0
Vilamin Mix#300050	3.9	10	39
Choline Bitartrate	0	2	0
Blue Dye	0	0.01	0
Yellow Dye	0	0.04	0

Table S2. High-fat mouse chow with 60% kcal from fat.

	gm%	Kcal%
Protein	26	20
Carbohydrate	26	20
Fat	35	60
Kcal/gm	5.26	

Ingredient	Caloric Value Per Unit (kcal/g)	Weight (gram)	Calories (kcal)
Caisein	4	200	800
L-Cystine	4	3	12
Sucrose	4	68.8	275
Dyetrose	4	125	500
Lard	9	245	2205
Soybean Oil	9	25	225
Cellulose	0	50	0
Mineral Mix#210088	1.6	10	16
Calcium Carbonate	0	5.5	0
Dicalcium Phosphate	0	13	0
Potassium Citrate H20	0	16.5	0
Vilamin Mix#300050	3.9	10	39
Choline Bitartrate	0	2	0
Blue Dye	0	0.05	0

References

1. Coia, H.; Ma, N.; Hou, Y.; Dyba, M.D.; Fu, Y.; Cruz, M.I.; Benitez, C.; Graham, G.T.; McCutcheon, J.N.; Zheng, Y.L.; et al, Prevention of Lipid Peroxidation-derived Cyclic DNA Adduct and Mutation in High Fat Diet-induced Hepatocarcinogenesis by Theaphenon E. *Cancer Prev. Res.* **2018**, *11*, 665–676. https://doi.org/10.1158/1940-6207.CAPR-18-0160.
2. Guo, J.; Pereira, T.J.; Dalvi, P.; Yeung, L.S.N.; Swain, N.; Breen, D.M.; Lam, L.; Dolinsky, V.W.; Giacca, A. High-dose metformin (420 mg/kg daily p.o.) increases insulin sensitivity but does not affect neointimal thickness in the rat carotid balloon injury model of restenosis. *Metabolism* **2016**, *68*, 108–118. https://doi.org/10.1016/j.metabol.2016.12.002.
3. Norris, G.H.; Porter, C.M.; Jiang, C.; Millar, C.L.; Blesso, C.N. Dietary sphingomyelin attenuates hepatic steatosis and adipose tissue inflammation in high-fat-diet-induced obese mice. *J. Nutr. Biochem.* **2017**, *40*, 36–43. https://doi.org/10.1016/j.jnutbio.2016.09.017.
4. Tordoff, M.G.; Aleman, T.R.; Murphy, M.C. No effects of monosodium glutamate consumption on the body weight or composition of adult rats and mice. *Physiol. Behav.* **2012**, *107*, 338–345. https://doi.org/10.1016/j.physbeh.2012.07.006.
5. Lee, Y.J.; Ko, E.H.; Kim, J.E.; Kim, E.; Lee, H.; Choi, H.; Yu, J.H.; Kim, H.J.; Seong, J.K.; Kim, K.S.; et al. Nuclear receptor PPARγ-regulated monoacylglycerol O-acyltransferase 1 (MGAT1) expression is responsible for the lipid accumulation in diet-induced hepatic steatosis. *Proc. Natl. Acad. Sci. USA* **2012** *109*, 13656–13661. https://doi.org/10.1073/pnas.1203218109.
6. Perry, R.J.; Resch, J.M.; Douglass, A.M.; Madara, J.C.; Rabin-Court, A.; Kucukdereli, H.; Wu, C.; Song, J.D.; Lowell, B.B.; Shulman, G.I. Leptin's hunger-suppressing effects are mediated by the hypothalamic–pituitary–adrenocortical axis in rodents. *ESPE Yearb. Paediatr. Endocrinol.* **2020**, *17*. https://doi.org/10.1530/ey.17.11.6.
7. Miranda, C.L.; Johnson, L.A.; de Montgolfier, O.; Elias, V.D.; Ullrich, L.S.; Hay, J.J.; Paraiso, I.L.; Choi, J.; Reed, R.L.; Revel, J.S.; et al. Non-estrogenic Xanthohumol Derivatives Mitigate Insulin Resistance and Cognitive Impairment in High-Fat Diet-induced Obese Mice. *Sci. Rep.* **2018**, *8*, 1–17. https://doi.org/10.1038/s41598-017-18992-6.
8. Mitchell, S.J.; Bernier, M.; Aon, M.A.; Cortassa, S.; Kim, E.Y.; Fang, E.F.; Palacios, H.H.; Ali, A.; Navas-Enamorado, I.; Di Francesco, A.; et al. Nicotinamide Improves Aspects of Healthspan, but Not Lifespan, in Mice. *Cell Metab.* **2018**, *27*, 667–676.e4. https://doi.org/10.1016/j.cmet.2018.02.001.
9. Kopec, A.K.; Abrahams, S.R.; Thornton, S.; Palumbo, J.S.; Mullins, E.S.; Divanovic, S.; Weiler, H.; Owens, A.P., 3rd.; Mackman, N.; Goss, A.; et al. Thrombin promotes diet-induced obesity through fibrin-driven inflammation. *Journalof Clin. Investig.* **2017**, *127*, 3152–3166. https://doi.org/10.1172/JCI92744.
10. Yang, J.W.; Kim, H.S.; Im, J.H.; Kim, J.W.; Jun, D.W.; Lim, S.C.; Lee, K.; Choi, J.M.; Kim, S.K.; Kang, K.W. GPR119: A promising target for nonalcoholic fatty liver disease. *FASEB J.* **2016**, *30*, 324–335. https://doi.org/10.1096/fj.15-273771.
11. Miyao, M.; Kotani, H.; Ishida, T.; Kawai, C.; Manabe, S.; Abiru, H.; Tamaki, K. Pivotal role of liver sinusoidal endothelial cells in NAFLD/NASH progression. *Mod. Pathol.* **2015**, *95*, 1130–1144. https://doi.org/10.1038/labinvest.2015.95.

Disclaimer/Publisher's Note: The statements, opinions and data contained in all publications are solely those of the individual author(s) and contributor(s) and not of MDPI and/or the editor(s). MDPI and/or the editor(s) disclaim responsibility for any injury to people or property resulting from any ideas, methods, instructions or products referred to in the content.
