# Supplementary material for: Ginsenoside Rg5 Activates the LKB1/AMPK/mTOR Signaling Pathway and Modifies the Gut Microbiota to Alleviate Nonalcoholic Fatty Liver Disease Induced by a High-Fat Diet
Source: Nutrients. 2024 Mar 15;16(6):842. doi: 10.3390/nu16060842 (PMC10974897; doi:10.3390/nu16060842)
Supplement: Supplementary file 1 [file Supplementary file Figure S1 and S2.pdf › Supplementary file Figure S1 and S2.pdf]

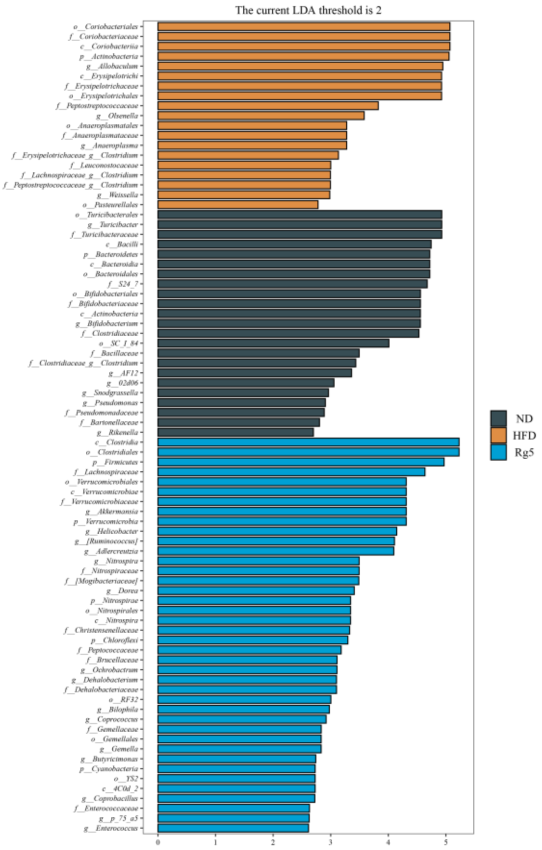

Figure S1. LDA effect size analysis effect size analysis of characteristic genera of the gut microbiota (LEfSe).

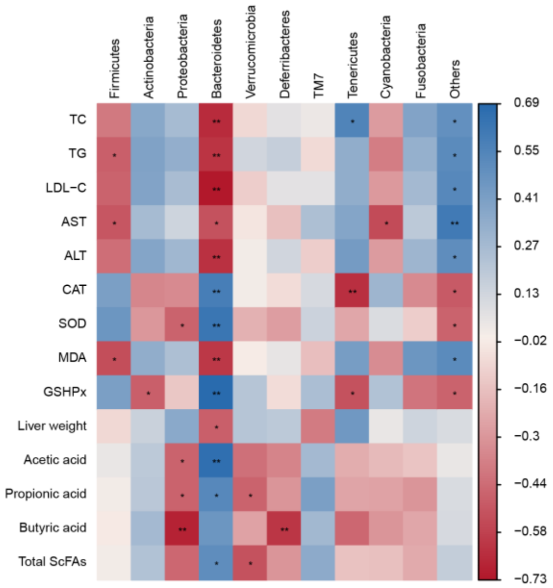

Figure S2. Heat map showing the Spearman correlation between glycolipid metabolism index, levels of SCFAs, and microorganisms at the phylum and genus levels in four groups of mice, respectively. Blue in-dicates high values, while red indicates low values. n=6, significant correlations are indicated by * $p < 0.05$ and ** $p < 0.01$.
